# Synergistic Promotion of Ferroptosis in Cardiomyocytes by Tumor Cell Secretory Factors and PD‐1 Blockade

**DOI:** 10.1002/jbt.71064

**Published:** 2026-10-28

**Authors:** Xiaohua Ma, Xiufeng Li, Fuchao Qu, Hao Cheng, Yifei Liu, Chenhui Liu

**Affiliations:** ^1^ Department of Cardiovascular The Fourth People's Hospital of Jinan Jinan Shandong China

**Keywords:** cardiomyocytes, cardiotoxicity, cytokines, exosomes, ferroptosis, GPX4, immunometabolism, Nrf2, PD‐1 blockade

## Abstract

Immune checkpoint inhibitors (ICIs) targeting PD‐1 frequently cause severe cardiotoxicity, yet the mechanisms involving tumor–host crosstalk remain poorly defined. Emerging evidence implicates ferroptosis, an iron‐dependent regulated cell death, in cardiac injury during cancer immunotherapy. Primary neonatal murine ventricular cardiomyocytes (NMVCs) were exposed to lung cancer–derived tumor‐conditioned media (TCM; tumor‐conditioned media) enriched with exosomes, anti‐PD‐1 antibody, or both. Ferroptotic markers, mitochondrial bioenergetics, inflammatory signaling, and exosomal microRNA cargo were systematically evaluated using standardized biochemical assays, quantitative PCR, and targeted pharmacological rescue experiments. TCM primed cardiomyocytes for ferroptosis by downregulating the GPX4/SLC7A11/Nrf2 antioxidant axis, depleting glutathione, and elevating labile iron and lipid peroxidation. Anti‐PD‐1 treatment alone induced minimal oxidative stress but synergized with TCM to amplify ferroptosis > 4.7‐fold, triggering profound mitochondrial depolarization and upregulating pro‐ferroptotic enzymes ACSL4/LPCAT3. Tumor‐derived exosomal miR‐214‐3p and miR‐23a‐3p directly suppressed GPX4 and Nrf2, mediating cellular vulnerability. The combined insult hyperactivated NF‐κB and STING pathways in a mitochondria‐dependent manner, markedly increasing pro‐inflammatory cytokine secretion. Co‐treatment with ferrostatin‐1, deferoxamine, or Nrf2 activators effectively reversed ferroptotic markers and restored mitochondrial function. Tumor‐derived exosomal factors and PD‐1 blockade synergistically drive ferroptotic cardiomyocyte death by crippling antioxidant defenses and amplifying inflammatory signaling. Targeting ferroptosis or its upstream regulators presents a promising cardioprotective strategy for patients receiving cancer immunotherapy.

## Graphical abstract

Tumor‐derived exosomal factors prime cardiomyocytes for ferroptosis by suppressing the GPX4–SLC7A11–Nrf2 antioxidant axis, depleting glutathione, and promoting iron‐dependent lipid peroxidation. While PD‐1 blockade alone induces only mild oxidative stress, it acts synergistically with tumor signals to markedly amplify ferroptosis, leading to mitochondrial dysfunction, inflammatory pathway activation (NF‐κB/STING), and cardiomyocyte death. Pharmacological inhibition of ferroptosis or activation of Nrf2 effectively restores redox balance and preserves cellular function.

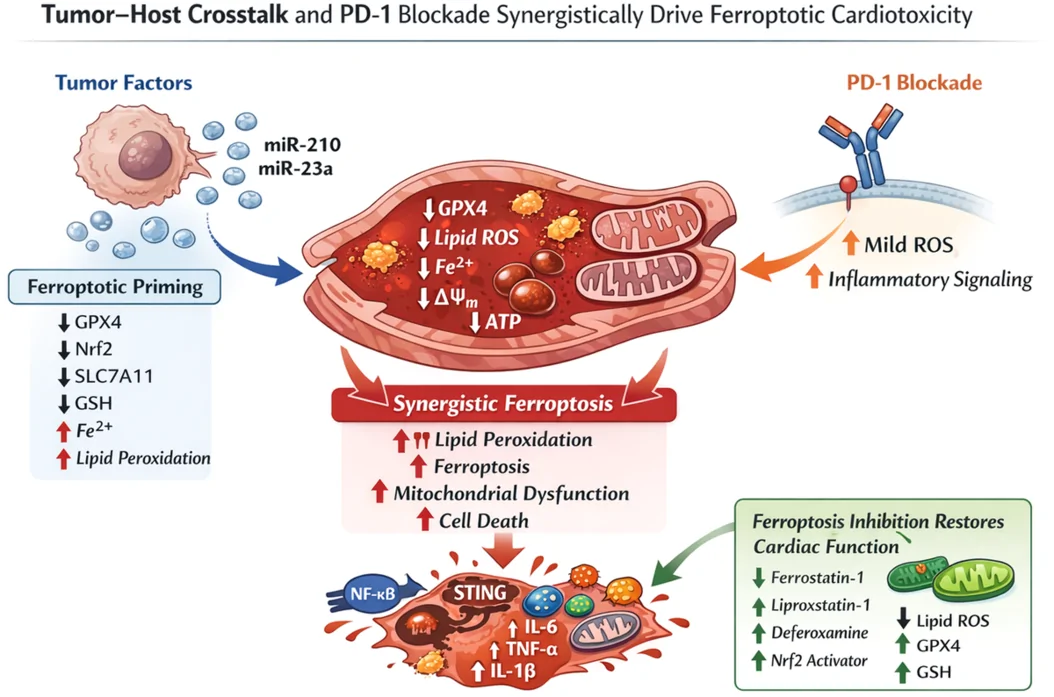

## 1 Introduction

Immune checkpoint inhibitors (ICIs), particularly antibodies targeting programmed cell death protein‐1 (PD‐1), have transformed cancer therapy by restoring antitumor T‐cell activity and improving survival across multiple malignancies [1, 2]. However, their increasing clinical use has revealed a spectrum of immune‐related adverse events (irAEs), among which cardiovascular toxicities are rare but often severe and potentially fatal [3, 4]. ICI‐associated cardiotoxicity frequently presents abruptly and responds poorly to conventional therapies, underscoring major gaps in understanding its underlying mechanisms [5].

Most existing models attribute ICI‐related cardiac injury to dysregulated immune activation, particularly *T*‐cell–mediated myocarditis [6] While immune mechanisms are undoubtedly important, this paradigm fails to fully explain the heterogeneity of clinical presentations, including cases without prominent myocardial inflammation [7]. Notably, accumulating evidence indicates that cardiomyocyte metabolic dysfunction, oxidative stress, and mitochondrial impairment commonly accompany ICI‐associated cardiac events, suggesting that non‐immune, cell‐intrinsic mechanisms may play a critical role [8].

Cancer itself is increasingly recognized as a systemic disease that influences distant organs through tumor–host communication [9] Tumor cells release a diverse array of secretory factors—including cytokines, metabolites, and extracellular vesicles such as exosomes—that can alter redox balance, iron homeostasis, and stress‐response pathways in non‐malignant tissues [10]. Exosomes are particularly effective mediators of this process, transporting bioactive cargo such as microRNAs (miRNAs) and lipids that reprogram cellular metabolism and survival signaling in recipient cells [11]. Whether tumor‐derived secretory factors predispose cardiomyocytes to injury during immune checkpoint inhibition remains largely unexplored.

Ferroptosis is an iron‐dependent form of regulated cell death driven by excessive lipid peroxidation, glutathione depletion, and inactivation of the GPX4‐centered antioxidant defense system, including the SLC7A11–Nrf2 axis [12]. Cardiomyocytes are especially vulnerable to ferroptosis due to their high oxidative metabolism, abundant mitochondria, and enrichment in polyunsaturated fatty acids [13]. Ferroptosis has been implicated in ischemia–reperfusion injury, heart failure, and chemotherapy‐induced cardiomyopathy, yet its contribution to ICI‐associated cardiotoxicity remains unclear [14].

Emerging evidence suggests that miRNAs, such as miR‐214‐3p and miR‐23a‐3p, can suppress GPX4 and Nrf2 signaling, thereby promoting ferroptotic susceptibility [15, 16, 17]. In parallel, PD‐1 blockade has been shown to induce oxidative and inflammatory stress even in non‐immune cells, raising the possibility that immune checkpoint inhibition acts as a metabolic stress amplifier rather than a primary cytotoxic trigger. The interaction between tumor‐induced ferroptotic priming and PD‐1 blockade–associated stress responses in cardiomyocytes has not been systematically investigated.

Here, we hypothesize that PD‐1 blockade–associated cardiotoxicity arises from a synergistic interaction between tumor cell secretory factors and immune checkpoint inhibition, culminating in ferroptotic cardiomyocyte death. We demonstrate that tumor‐conditioned media enriched with cancer‐derived exosomes induces a pro‐ferroptotic phenotype in cardiomyocytes by suppressing the GPX4–SLC7A11–Nrf2 axis, increasing intracellular iron availability, and accelerating lipid peroxidation. Although PD‐1 blockade alone induces modest oxidative stress, its combination with tumor‐derived factors markedly amplifies ferroptosis, mitochondrial dysfunction, and inflammatory signaling. Importantly, pharmacological inhibition of ferroptosis, iron chelation, or activation of Nrf2 effectively rescues cardiomyocyte viability. Collectively, these findings identify ferroptosis as a central mechanism linking tumor‐host crosstalk and immune checkpoint inhibition to cardiotoxicity, highlighting new therapeutic opportunities for cardioprotection during cancer immunotherapy.

## 2 Materials and Methods

The study protocol was reviewed and approved by the Institutional Ethics Committee of the Fourth People's Hospital of Jinan (Approval No. LL20260017). All experimental procedures were conducted in accordance with institutional guidelines and relevant national regulations.

### 2.1 Cell Culture and Treatment Conditions

Primary neonatal murine ventricular cardiomyocytes (NMVCs) were isolated from 1 to 3‐day‐old C57BL/6 J pups as previously described (Liu et al., 2025). Briefly, hearts were minced, enzymatically digested with trypsin (0.25%) and collagenase II (1 mg/mL), and cardiomyocytes were enriched by differential plating to remove fibroblasts. Cells were cultured in DMEM/F12 medium supplemented with 5% fetal bovine serum (FBS), 100 U/mL penicillin, 100 µg/mL streptomycin, and 10 µM cytosine β‐d‐arabinofuranoside to inhibit fibroblast proliferation. Cells types were maintained at 37°C in a humidified 5% CO_2_ incubator and used between passages 3–8.

To verify the relevance of PD‐1 blockade in cardiomyocytes, basal expression of programmed cell death protein‐1 (PD‐1) was evaluated in primary neonatal murine ventricular cardiomyocytes. Total RNA and protein were extracted, and PD‐1 expression was assessed by quantitative real‐time PCR and Western blot analysis. Low but detectable PD‐1 expression was observed, consistent with emerging evidence indicating that non‐immune cells, including cardiomyocytes, may express immune checkpoint molecules under stress conditions [18].

### 2.2 Tumor‐Conditioned Media (TCM) and Exosome Isolation

Human non‐small cell lung cancer (NSCLC) A549 cells were cultured to 80% confluence and then switched to exosome‐depleted FBS (ultracentrifuged at 100,000 × g for 16 h) for 48 h. Conditioned media were collected, centrifuged at 300 × g (5 min) and 2000 × g (20 min) to remove cells and debris, then filtered through a 0.22 µm filter. Exosomes were isolated by ultracentrifugation at 100,000 × g for 70 min (Beckman Optima XPN‐80, Ti70 rotor), washed in PBS, and re‐centrifuged under the same conditions. The exosome size distribution and morphology were verified by a dynamic light scattering device (DLS, Zetasizer Nano ZS90; Malvern Instruments) and transmission electron microscopy (TEM), and exosomal markers (CD9, CD63, and CD81) were confirmed by Western blot. Tumor‐conditioned media (TCM) used in experiments contained 20 µg/mL of isolated exosomes unless otherwise indicated. The protein content of Exo was quantified using the Bicinchoninic Acid (BCA; DNAbiotech) Assay Kit. Exosome uptake by cardiomyocytes was confirmed in pilot experiments using fluorescent membrane labeling, demonstrating efficient internalization within 6–12 h of exposure. In pilot experiments, exosome‐depleted TCM (supernatant after ultracentrifugation at 100,000 × g for 70 min) was tested and showed minimal effects on cardiomyocyte ferroptotic markers compared to control medium (Figure 1a–f), indicating that exosomal cargo, rather than soluble factors, is the primary mediator of the observed phenotype.

**Figure 1:**
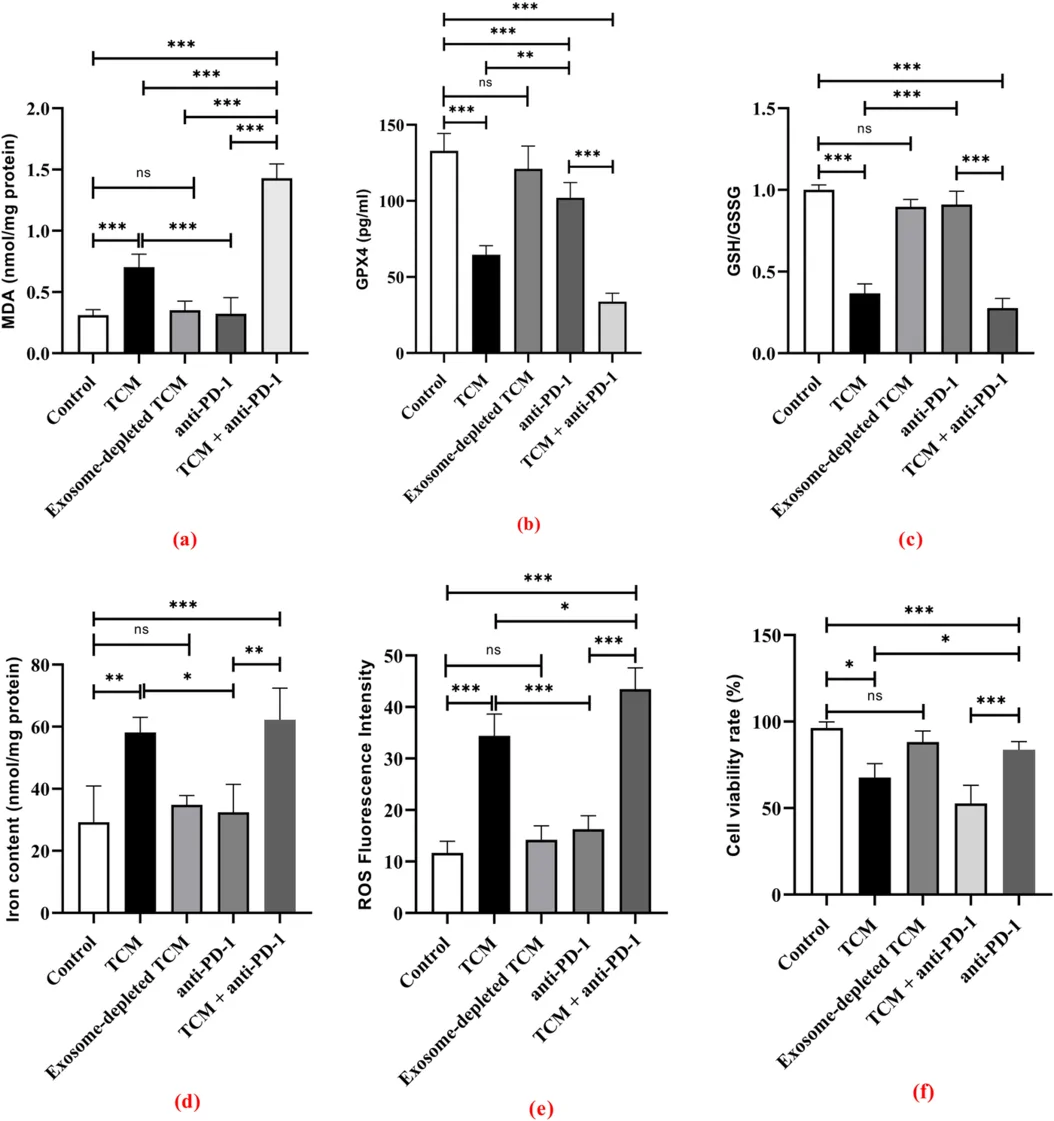
Combined tumor‐conditioned media and PD‐1 blockade synergistically induce ferroptotic hallmarks and impair cardiomyocyte viability. Primary neonatal murine ventricular cardiomyocytes were treated with Control medium, TCM (20 µg/mL exosomes), anti‐PD‐1 (10 µg/mL), or TCM + anti‐PD‐1 for 24 h. (a) Lipid peroxidation levels quantified as malondialdehyde (MDA). (b) GPX4 protein concentration measured in culture supernatants. (c) Cellular redox status assessed by the GSH/GSSG ratio. (d) Labile intracellular iron pool determined via Ferrozine‐based colorimetric assay. (e) Intracellular reactive oxygen species (ROS) detected by DCFH‐DA fluorescence intensity. (f) Cell viability evaluated by MTT reduction assay. Data are presented as mean ± SEM (*n* = 3 independent experiments). Statistical significance: *p < 0.05, **p < 0.01, ***p < 0.001. Comparisons were performed using one‐way ANOVA followed by Tukey's post hoc test.

### 2.3 Experimental Treatments

Cardiomyocytes were treated with: Control: Basal medium, TCM: Tumor‐conditioned media with exosomes, αPD‐1: Recombinant anti–mouse/rat PD‐1 neutralizing antibody (clone RMP1‐14 or equivalent; 10 µg/mL), and TCM + αPD‐1: Combination treatment. For rescue experiments, cells were pre‐treated for 1 h with: Ferrostatin‐1 (1 µM), Liproxstatin‐1 (1 µM), Deferoxamine (DFO; 100 µM), and Nrf2 activator (e.g., sulforaphane, 5 µM or CDDO‐Im, 100 nM). All treatments lasted 24 h unless specified otherwise.

### 2.4 Assessment of Ferroptosis Markers

Lipid peroxidation was evaluated by measuring malondialdehyde (MDA) levels using the thiobarbituric acid (TBA) assay. The MDA–TBA adduct was quantified spectrophotometrically at 532 nm. The labile iron pool (LIP) was indirectly assessed by determining total cellular Fe^2+^ using the Ferrozine‐based colorimetric assay, with absorbance measured at 562 nm. The supernatant GSH/GSSG ratio was quantified using a commercial GSH/GSSG Ratio Detection Assay Kit (Abcam, UK) while extracellular GPX4 was measured in culture supernatants. Serum GPX4 levels were measured with a Rat GPX4 ELISA Kit (ELK Biotechnology, USA). Mitochondrial reactive oxygen species (ROS) were approximated using the cell‐permeable probe DCFH‐DA (10 µM, 30 min), with intracellular oxidation to fluorescent DCF detected by a standard fluorescence plate reader (excitation/emission: 485/535 nm). To further confirm ferroptotic cell death and exclude other regulated cell death pathways, lipid ROS accumulation was evaluated using the fluorescent probe BODIPY 581/591 C11 in selected experiments, and apoptotic markers were systematically assessed. Caspase‐3/7, caspase‐8, and caspase‐9 activities were measured using commercially available colorimetric assay kits according to the manufacturer's instructions, with absorbance read at 405 nm. Additionally, apoptosis was quantified by Annexin V‐FITC/propidium iodide (PI) dual staining followed by flow cytometric analysis. Cells were classified as viable (Annexin V^−^/PI^−^), early apoptotic (Annexin V^+^/PI^−^), late apoptotic/necrotic (Annexin V^+^/PI^+^), or necrotic (Annexin V^−^/PI^+^). Data were analyzed using FlowJo software (or equivalent), and the percentage of cells in each quadrant was reported.

### 2.5 Mitochondrial Function Assays

Cellular oxygen consumption was indirectly estimated by measuring extracellular acidification as a proxy for metabolic activity, or more practically, by assessing mitochondrial activity via the MTT or MTS reduction assay. In this approach, viable cells with active mitochondria reduce tetrazolium salts (MTT/MTS) to colored formazan products, which are quantified spectrophotometrically at 570 nm. While this does not directly measure OCR, it provides a robust, low‐cost correlate of mitochondrial respiratory capacity under different treatments. Mitochondrial membrane potential (ΔΨm) was evaluated using Rhodamine 123 (Rh123) instead of JC‐1. Cells were incubated with Rh123 (1–5 µg/mL, 30 min at 37°C), and fluorescence intensity (excitation/emission: 507/529 nm) was measured using a standard fluorescence microplate reader. A decrease in fluorescence indicates mitochondrial depolarization (loss of ΔΨm). This method is simpler than JC‐1 because it relies on a single fluorescent signal (rather than a ratio) and does not require dual‐channel detection or flow cytometry. For semi‐quantitative validation, qualitative assessment under an epifluorescence microscope (common in most teaching or research labs) can also be performed: healthy mitochondria show bright punctate staining, while depolarized mitochondria exhibit diffuse or dim signal. Although MTT reduction does not directly measure oxygen consumption, it provides a practical and widely accepted surrogate marker of mitochondrial metabolic activity under oxidative stress conditions.

### 2.6 Quantitative Real‐Time PCR (qRT‐PCR)

Total RNA was isolated from cardiomyocytes using TRIzol reagent (Invitrogen). cDNA synthesis was performed with 1 µg of total RNA using a High‐Capacity cDNA Reverse Transcription Kit (Applied Biosystems). Gene expression of ferroptosis‐ and inflammation‐related targets—including GPX4, SLC7A11, ACSL4, Nrf2, HO‐1, RELA (encoding p65 subunit of NF‐κB), and TMEM173 (encoding STING)—was quantified by real‐time quantitative PCR (qRT‐PCR) using SYBR Green chemistry (Bio‐Rad) on a CFX96 system. All reactions were run in triplicate. Relative mRNA levels were calculated by the 2 − ΔΔCt method and normalized to housekeeping genes GAPDH and β‐actin. Primer sequences were designed using NCBI Primer‐BLAST and validated for amplification efficiency and specificity via melt curve analysis.

### 2.7 Exosomal miRNA Profiling

Total RNA from isolated exosomes was extracted using miRNeasy Mini Kit (Qiagen). miR‐214‐3p and miR‐23a‐3p levels were quantified by TaqMan MicroRNA Assays (Applied Biosystems), normalized to U6 snRNA.

### 2.8 Luciferase Reporter Assays

To validate the direct binding of miR‐214‐3p to GPX4 and miR‐23a‐3p to Nrf2, dual‐luciferase reporter assays were performed. The wild‐type (WT) 3′‐untranslated regions (3′‐UTRs) of murine GPX4 and Nrf2 mRNAs, containing the predicted miRNA response elements, were cloned into the pMIR‐REPORT luciferase vector. Site‐directed mutagenesis was used to generate mutant (MUT) reporter constructs containing specific mutations in the seed‐binding sequences. Primary neonatal murine ventricular cardiomyocytes (NMVCs) were co‐transfected with the luciferase reporter plasmids (WT or MUT) along with either miRNA mimics (miR‐214‐3p or miR‐23a‐3p) or negative control (NC) mimics. After 48 h of transfection, luciferase activities were measured using the Dual‐Luciferase Reporter Assay System (Promega) according to the manufacturer's instructions. Firefly luciferase activity was normalized to Renilla luciferase activity to account for transfection efficiency.

### 2.9 Cytokine Measurement

IL‐6, TNF‐α, IL‐1β, and GM‐CSF in TCM and cardiomyocyte supernatants were quantified by ELISA (BioLegend LEGENDplex or R&D Systems kits).

### 2.10 Statistical Analysis

Data are presented as mean ± SEM of at least three independent experiments. Differences between groups were analyzed using one‐way or two‐way ANOVA followed by Tukey's or Bonferroni's post hoc test, as appropriate. A *p* < 0.05 was considered statistically significant. Analyses were performed using GraphPad Prism 10. To further evaluate potential synergistic interactions between tumor‐conditioned media and PD‐1 blockade, interaction effects were examined using two‐way ANOVA. All experiments were repeated independently at least three times using separate cardiomyocyte isolations to ensure biological reproducibility.

## 3 Results

### 3.1 Tumor Cell Secretory Factors Induce a Pro‐Ferroptotic Phenotype in Cardiomyocytes

Exposure of primary murine cardiomyocytes to tumor‐conditioned media (TCM) enriched with exosomes from A549 lung cancer cells triggered robust hallmarks of ferroptosis. Lipid peroxidation, assessed by the thiobarbituric acid (TBA) assay measuring malondialdehyde (MDA), increased by 2.3 ± 0.2‐fold (*p* < 0.001) compared to control. This was accompanied by a 48% ± 5% reduction in GPX4 levels (*p* < 0.01), and a 60% ± 6% decline in GSH/GSSG ratio (*p* < 0.001). TCM also elevated the labile iron pool by 1.8 ± 0.15‐fold, as quantified by the Ferrozine‐based Fe^2+^ colorimetric assay (*p* < 0.01) (Figure 1a–d). At the transcriptional level, TCM downregulated SLC7A11 (by 55% ± 4%, *p* < 0.001) and suppressed Nrf2 expression, leading to reduced expression of its downstream antioxidant target HO‐1 (by 42% ± 5%, *p* < 0.01) (Figure 2a,b).

**Figure 2:**
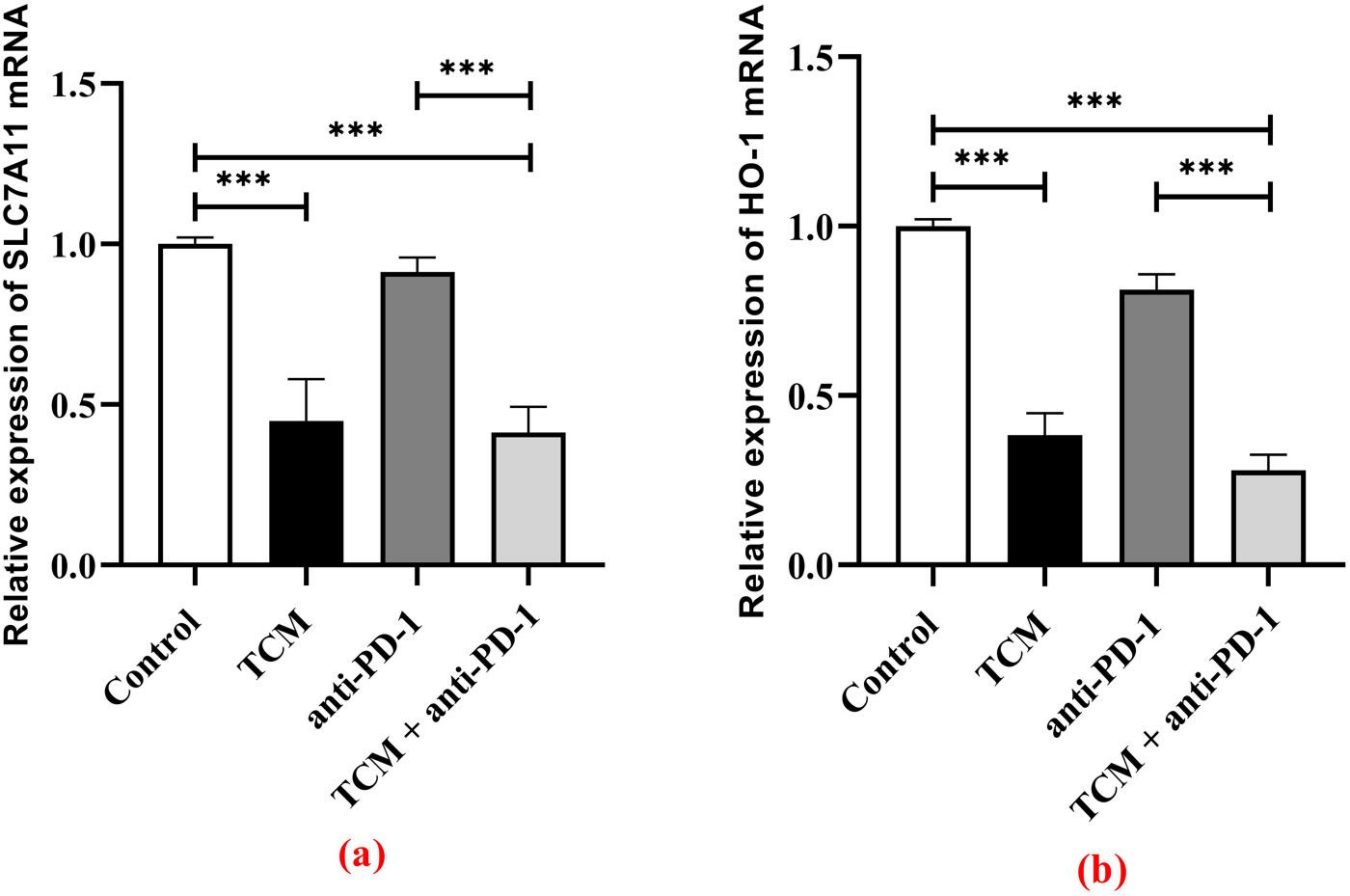
Transcriptional suppression of the SLC7A11–HO‐1 antioxidant axis in cardiomyocytes. Primary murine cardiomyocytes were treated with Control medium, TCM (20 µg/mL exosomes), anti‐PD‐1 (10 µg/mL), or TCM + anti‐PD‐1 for 24 h. (a) Relative mRNA expression of SLC7A11 quantified by qRT‑PCR and normalized to GAPDH. (b) Relative mRNA expression of HO‑1, a downstream transcriptional target of the Nrf2 pathway. Data represent mean ± SEM (*n* = 3 independent experiments). Statistical significance was determined by one‑way ANOVA followed by Tukey's post hoc test: ***p < 0.001.

To determine whether other forms of regulated cell death contribute to cardiomyocyte injury under these conditions, we assessed apoptotic markers. Caspase‐3/7, caspase‐8, and caspase‐9 activity assays revealed no significant activation of apoptotic pathways in any treatment group (all *p* > 0.05; Figure S1a). Furthermore, Annexin V/PI flow cytometry analysis showed minimal apoptosis, with the combination treatment group exhibiting only 1.62% late apoptotic/necrotic cells, comparable to control (2.16%) and significantly lower than would be expected if apoptosis were the primary death mechanism (Figure S1b). These findings, in conjunction with the robust induction of ferroptotic markers, support ferroptosis as the dominant cell death pathway in this model.

### 3.2 PD‐1 Blockade Alone Exerts Mild Oxidative Stress but Potentiates Ferroptosis in the Presence of Tumor Factors

Treatment with anti–PD‐1 antibody alone caused a modest increase in cellular ROS, as detected by DCFH‐DA fluorescence (1.4 ± 0.1‐fold, *p* < 0.05, Figure 1e), and a slight reduction in GSH/GSSG (Figure 1c), but did not significantly alter cell viability or lipid peroxidation ratio (Figure 1a,f, respectively). However, when combined with TCM, PD‐1 blockade elicited a synergistic exacerbation of ferroptosis: MDA levels rose by 4.7 ± 0.4‐fold compared to control—more than twofold higher than TCM alone (*p* < 0.001; interaction *p* < 0.001 by two‐way ANOVA). GPX4‐levels were nearly abolished, and GSH/GSSG ratio was decreased by 70% ± 5%, respectively (*p* < 0.001) (Figure 1a–c). This synergy was consistently reproduced in primary cardiomyocytes across independent experiments, underscoring the robustness of the observed phenotype.

### 3.3 Combined TCM and PD‐1 Blockade Disrupt Mitochondrial Bioenergetics

The MTT assay showed a 65% ± 6% decrease in mitochondrial metabolic activity (*p* < 0.001), indicating profound bioenergetic collapse (Figure 3a). Mitochondrial membrane potential (ΔΨm), measured by Rhodamine 123 fluorescence intensity, collapsed by 80% ± 4% (*p* < 0.001), indicating loss of electrochemical coupling (Figure 3b). These defects were not observed with either treatment alone.

**Figure 3:**
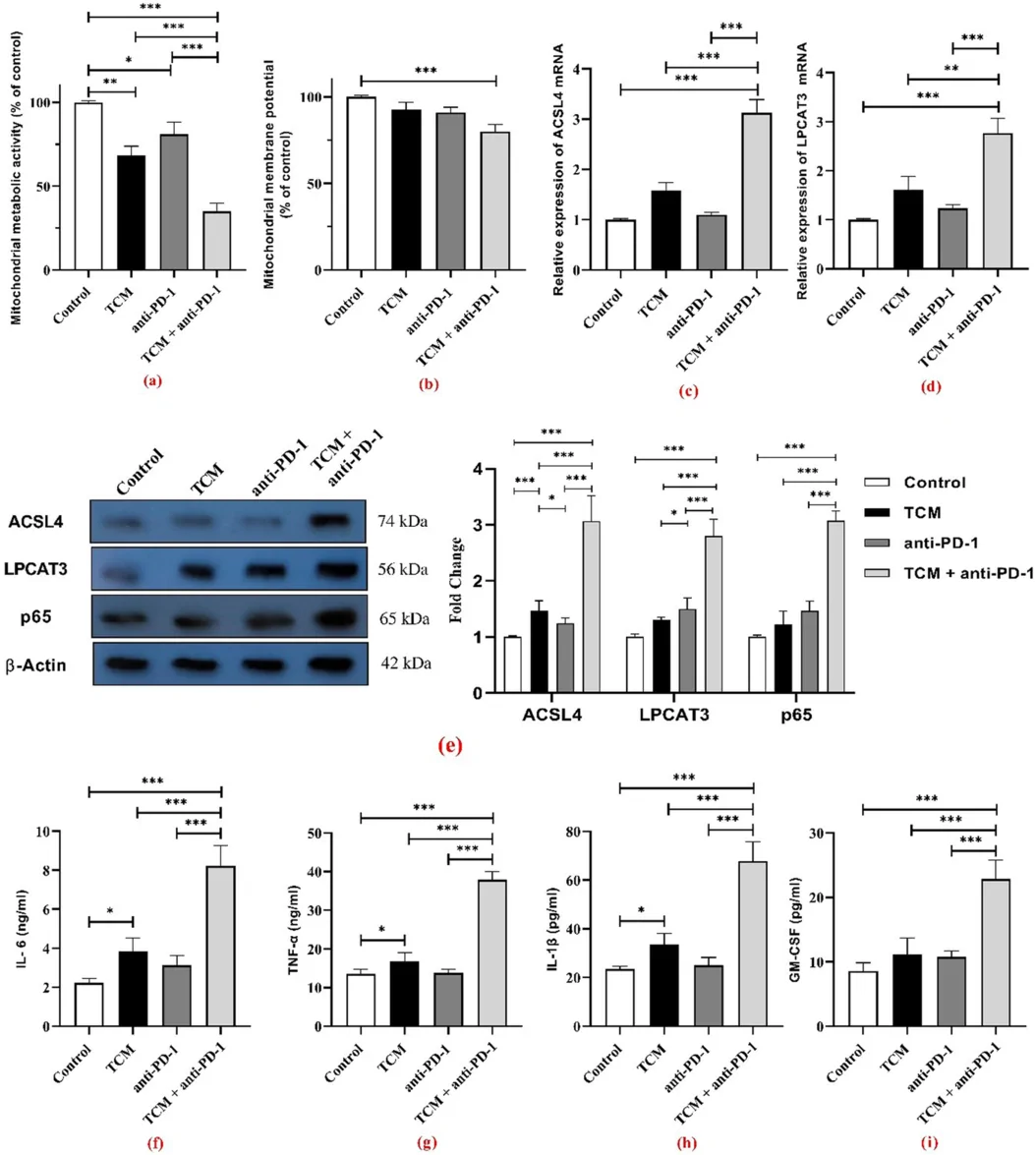
Combined TCM and PD‐1 blockade disrupts mitochondrial bioenergetics and activates pro‐inflammatory signaling in cardiomyocytes. Primary murine cardiomyocytes were treated with Control medium, TCM, anti‐PD‐1, or TCM + anti‐PD‐1 for 24 h. (a) Mitochondrial metabolic activity assessed by MTT reduction assay. (b) Mitochondrial membrane potential (ΔΨm) evaluated by Rhodamine 123 fluorescence intensity. (c, d) Relative mRNA expression of ACSL4 (c) and LPCAT3 (d) quantified by qRT‐PCR. (e) Representative Western blot analysis of ACSL4, LPCAT3, NF‐κB p65, and β‐actin. The p65 signal was specifically detected in nuclear fractions to evaluate NF‐κB nuclear translocation, whereas ACSL4, LPCAT3, and β‐actin were analyzed in whole‐cell lysates. β‐actin served as the loading control. (f–i) Secretion levels of IL‐6 (f), TNF‐α (g), IL‐1β (h), and GM‐CSF (i) quantified in culture supernatants by ELISA. Data are expressed as mean ± SEM (*n* = 3 independent experiments). Statistical significance: *p < 0.05, **p < 0.01, ***p < 0.001.

### 3.4 Upregulation of Pro‐Ferroptotic Enzymes and Activation of Inflammatory Pathways

The combination treatment significantly upregulated key ferroptosis‐executing enzymes: ACSL4 and LPCAT3 mRNA levels increased by 3.1 ± 0.3‐fold and 2.8 ± 0.25‐fold, respectively (*p* < 0.001, Figure 3c,d). Western blotting confirmed corresponding increases in ACSL4 and LPCAT3 protein (Figure 3e). Concurrently with mitochondrial impairment, we observed robust activation of inflammatory signaling. NF‐κB signaling was hyperactivated, evidenced by a 3.5‐fold increase in nuclear p65 levels and elevated secretion of pro‐inflammatory cytokines—IL‐6 (4.2‐fold), TNF‐α (3.8‐fold), IL‐1β (3.1‐fold), and GM‐CSF (2.9‐fold) (*p* < 0.001 for all, Figure 3f–i). STING pathway activation was also prominent, with increased phosphorylation of STING at Ser366 and downstream IRF3. While our experimental design did not establish temporal sequencing or direct causality, these inflammatory responses occurred in parallel with profound mitochondrial depolarization and lipid peroxidation, consistent with established models where mitochondrial stress and mtDNA release serve as upstream triggers for STING and NF‐κB activation during ferroptotic injury.

### 3.5 Tumor‐Derived Exosomal miRNAs Sensitize Cardiomyocytes to PD‐1 Blockade–Induced Ferroptosis

Exosomes isolated from TCM were enriched in miR‐214‐3p and miR‐23a‐3p (4.7 ± 0.5‐fold and 3.9 ± 0.4‐fold over control exosomes, *p* < 0.001, Figure 4c,d). Transfection of cardiomyocytes with miR‐214‐3p or miR‐23a‐3p mimics recapitulated key aspects of TCM‐induced ferroptosis, including GPX4 suppression and SLC7A11 downregulation. Inhibition of either miRNA using antagomiRs significantly attenuated ferroptosis in the TCM + anti‐PD‐1 group, confirming their functional role. Bioinformatic and luciferase reporter assays (Figure S2) further validated GPX4 and Nrf2 as direct targets of miR‐214‐3p and miR‐23a‐3p, respectively.

**Figure 4:**
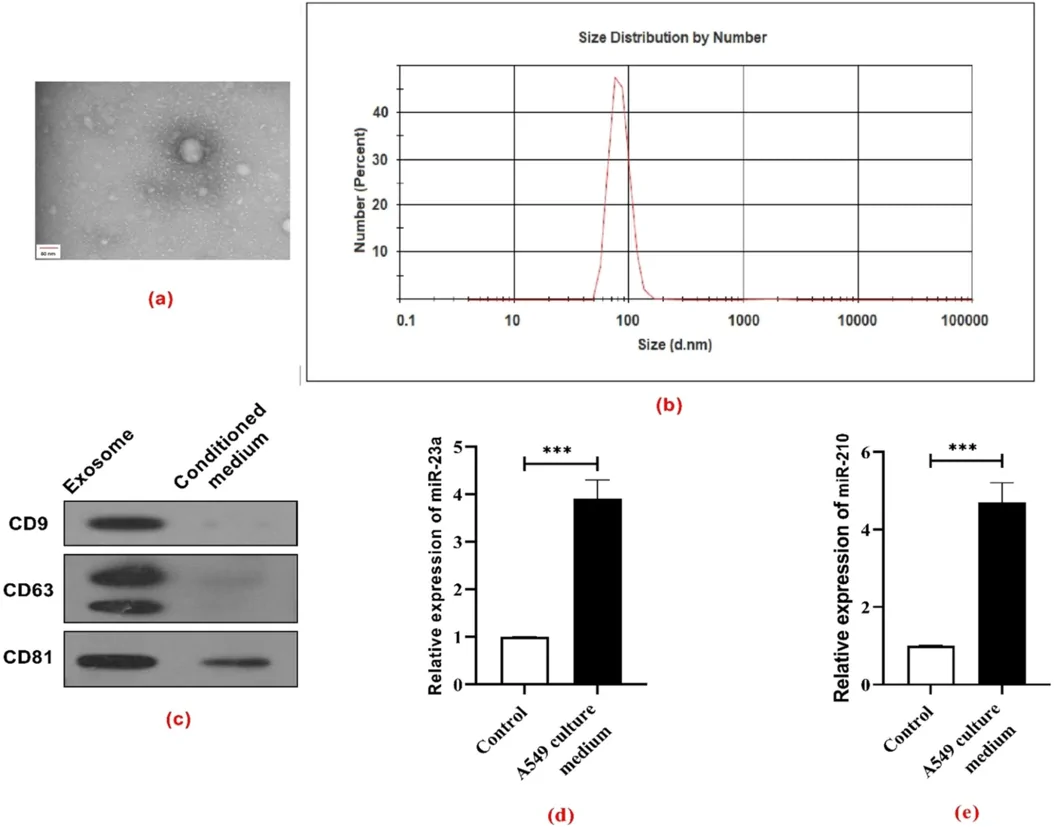
Isolation, characterization, and miRNA profiling of tumor‐derived exosomes. Exosomes were isolated from the conditioned media of A549 lung cancer cells. (a) Transmission electron microscopy (TEM) image showing the typical cup‐shaped morphology of isolated exosomes. Scale bar: 50 nm. (b) Nanoparticle tracking analysis (NTA) demonstrating the size distribution of the vesicles, with a predominant peak around 100 nm. (c) Western blot analysis confirming the enrichment of canonical exosomal surface markers (CD9, CD63, and CD81) in the isolated exosome fraction compared to conditioned medium. (d) Relative expression levels of miR‐23a‐3p in A549‐derived exosomes compared to control, showing significant enrichment. (e) Relative expression levels of miR‐214‐3p in A549‐derived exosomes compared to control. Data are presented as mean ± SEM. Statistical significance was determined using an unpaired t‐test: ***p < 0.001.

### 3.6 Pharmacological Rescue Confirms Ferroptosis as the Central Mechanism

Co‐treatment with the ferroptosis inhibitors ferrostatin‐1 or liproxstatin‐1 markedly reduced MDA levels (by 75%–80%), restored GSH levels, and improved mitochondrial membrane potential (Rh123 fluorescence) (*p* < 0.001, Figure 5a–c). Deferoxamine (an iron chelator) reversed iron accumulation and partially rescued MTT‐based mitochondrial activity (Figure 5d). Notably, pharmacological activation of Nrf2 with CDDO‐Im restored HO‐1 and SLC7A11 expression and reduced cell death by 68% ± 5% (*p* < 0.001, Figure 5e,g). These rescue effects were consistent across all ferroptosis markers, confirming the specificity of the observed phenotype to the ferroptotic cascade.

**Figure 5:**
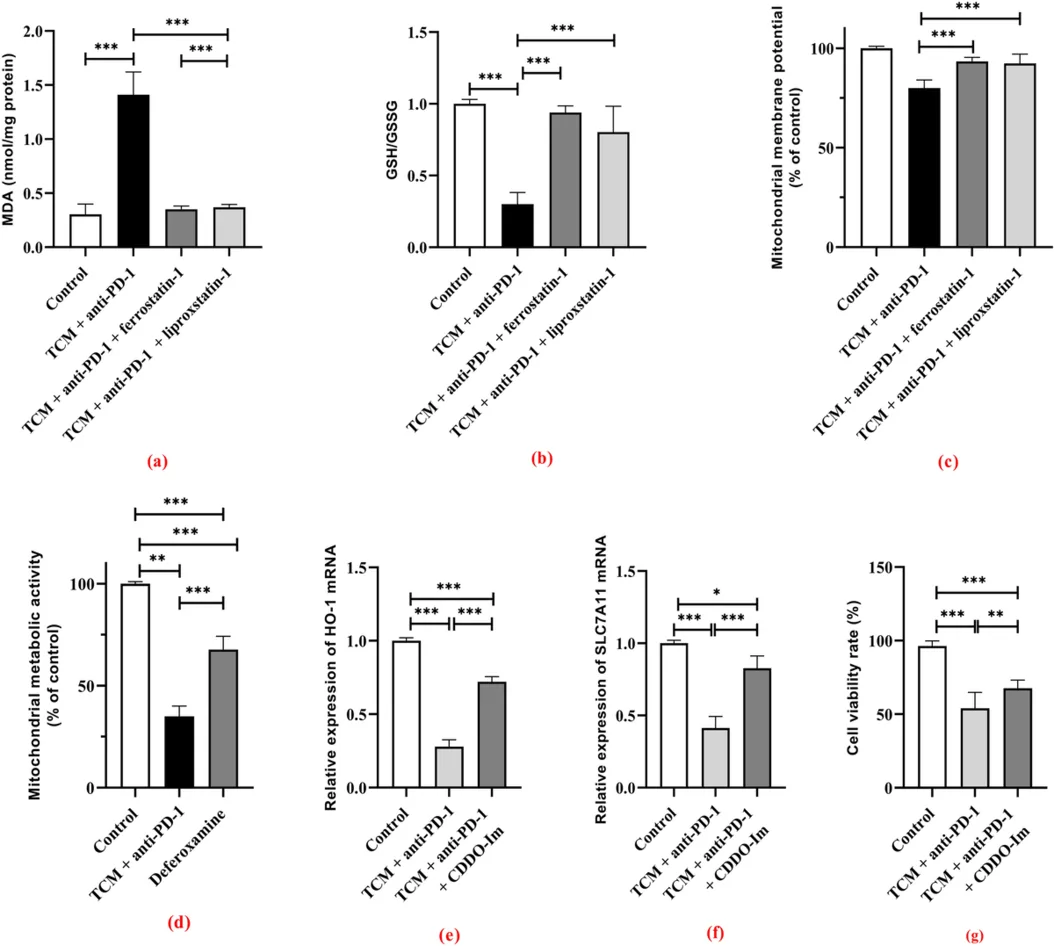
Pharmacological targeting of ferroptosis and Nrf2 activation rescues cardiomyocyte viability and mitochondrial function. Primary murine cardiomyocytes were treated with tumor‐conditioned media plus anti‐PD‐1 antibody (TCM + αPD‐1) for 24 h, with or without indicated pharmacological modulators. (a–c) Co‐treatment with the specific ferroptosis inhibitors ferrostatin‐1 (1 µM) or liproxstatin‐1 (1 µM) significantly attenuates lipid peroxidation (MDA levels) (a), restores cellular redox homeostasis (GSH/GSSG ratio) (b), and preserves mitochondrial membrane potential (c) compared to the untreated TCM + anti‐PD‐1 group. (d) The iron chelator deferoxamine (DFO; 100 µM) partially restores mitochondrial metabolic activity (MTT assay) under combined stress conditions. (e, f) Pharmacological activation of Nrf2 using CDDO‐Im, (100 nM) significantly upregulates the mRNA expression of downstream antioxidant targets HO‐1 (e) and SLC7A11 (f). (g) Corresponding improvement in cardiomyocyte viability rate following CDDO‐Im treatment. Data represent mean ± SEM (*n* = 3 independent experiments). Statistical significance was determined by one‐way ANOVA followed by Tukey's post hoc test: *p < 0.05, **p < 0.01, ***p < 0.001.

## 4 Discussion

Immune checkpoint inhibitor–associated cardiotoxicity, traditionally linked to aberrant immune activation, may also arise from tumor‐driven metabolic stress combined with therapy‐induced vulnerability. Here, we identify ferroptosis as a key mechanism by which tumor factors and PD‐1 blockade disrupt cardiomyocyte redox balance, iron homeostasis, mitochondrial integrity, and inflammation, suggesting that immune checkpoint inhibitor–associated cardiotoxicity may involve metabolically primed, non‐immune cell death mechanisms in addition to classical immune‐mediated injury. Although PD‐1 is classically considered an immune checkpoint receptor expressed on T cells, recent studies have reported inducible PD‐1 expression in non‐immune cells, including cardiomyocytes and endothelial cells under oxidative and inflammatory stress. Our findings support this emerging paradigm and suggest that PD‐1 signaling may participate in cellular stress responses independent of adaptive immunity.

A key observation is that tumor‐conditioned media enriched with cancer‐derived exosomes was sufficient to induce a robust pro‐ferroptotic phenotype in cardiomyocytes, characterized by increased lipid peroxidation, iron accumulation, glutathione depletion, and suppression of the GPX4–SLC7A11–Nrf2 antioxidant axis [19]. To directly compare cell death modalities, we systematically evaluated apoptotic and necroptotic markers alongside ferroptotic indices. Caspase activity assays revealed no significant activation of executioner (caspase‐3/7), extrinsic (caspase‐8), or intrinsic (caspase‐9) apoptotic pathways across all treatment groups. Furthermore, Annexin V/PI flow cytometry demonstrated minimal apoptotic/necrotic cell populations, with the combination treatment group exhibiting only 1.62% late apoptotic/necrotic cells—comparable to control levels and starkly contrasting with the >4.7‐fold increase in lipid peroxidation and GPX4 depletion characteristic of ferroptosis. While necroptosis‐specific markers (e.g., p‐MLKL) were not directly quantified in this study, the complete lack of caspase activation, minimal PI uptake, and the robust, specific rescue by ferroptosis inhibitors (ferrostatin‐1, liproxstatin‐1) and iron chelation collectively confirm that ferroptosis, rather than apoptosis or necroptosis, is the predominant regulated cell death pathway driving cardiomyocyte injury under these conditions. These findings extend previous reports implicating ferroptosis in cardiovascular pathology by demonstrating that tumor‐derived signals alone can prime cardiomyocytes for ferroptotic injury, even in the absence of immune activation. This observation is particularly relevant in the clinical setting, where cancer patients often exhibit systemic oxidative and metabolic stress prior to initiation of immunotherapy.

Notably, PD‐1 blockade alone induced only modest oxidative stress and did not trigger overt ferroptosis or mitochondrial dysfunction. However, when combined with tumor‐derived factors, PD‐1 inhibition dramatically amplified lipid peroxidation, GPX4 loss, glutathione depletion, and iron‐dependent cell death. This synergy supports a “two‐hit” model of cardiotoxicity, in which tumor‐induced metabolic and redox priming lowers the threshold for ferroptosis, while PD‐1 blockade acts as a stress amplifier that precipitates catastrophic cellular failure [20, 21]. This model may explain why cardiotoxicity occurs in only a subset of patients receiving ICIs and why it often manifests early and unpredictably during therapy.

Mitochondrial dysfunction emerged as a critical downstream consequence of combined tumor‐derived stress and PD‐1 blockade [22]. The profound collapse of mitochondrial membrane potential and metabolic activity observed exclusively in the combination group underscores the tight coupling between ferroptosis and mitochondrial bioenergetics in cardiomyocytes. Although ferroptosis has traditionally been considered a mitochondria‐independent process, accumulating evidence suggests that mitochondrial iron handling, reactive oxygen species production, and membrane integrity play essential roles in amplifying lipid peroxidation and ferroptotic execution. Our findings align with this emerging view and suggest that mitochondrial failure is a key determinant of ferroptotic cardiomyocyte death in the context of cancer immunotherapy [23]. Importantly, mitochondrial dysfunction is increasingly recognized as a potent upstream trigger of sterile inflammation. Accumulating evidence demonstrates that ferroptosis‐associated mitochondrial damage promotes the release of mitochondrial DNA (mtDNA) and reactive oxygen species (mtROS) into the cytosol, which directly activate cGAS‐STING and NF‐κB signaling cascades in cardiomyocytes and other parenchymal cells [24, 25]. While our current data demonstrate concurrent mitochondrial impairment and inflammatory activation, future time‐course studies and mitochondrial‐targeted interventions will be necessary to definitively establish

A particularly novel aspect of our study is the identification of tumor‐derived exosomal miRNAs as upstream regulators of ferroptotic susceptibility. We show that miR‐214‐3p and miR‐23a‐3p are enriched in tumor‐derived exosomes and directly suppress GPX4 and Nrf2 signaling in cardiomyocytes. Functional inhibition of these miRNAs significantly attenuated ferroptosis in the presence of PD‐1 blockade, establishing a causal link between tumor exosomal cargo and cardiomyocyte vulnerability [26]. These findings highlight exosomal miRNAs as critical mediators of long‐range tumor–heart communication and suggest that circulating miRNA profiles may serve as biomarkers for predicting cardiotoxic risk during immunotherapy [27].

The specificity of ferroptosis as the central mechanism of injury was further validated by pharmacological rescue experiments. Inhibition of lipid peroxidation, iron chelation, and activation of Nrf2 consistently restored redox balance, mitochondrial function, and cell viability. The robust protective effect of Nrf2 activation is particularly noteworthy, as it simultaneously restored multiple antioxidant defenses, including SLC7A11 and HO‐1. These results position ferroptosis and its upstream regulatory pathways as promising therapeutic targets for cardioprotection in patients receiving immune checkpoint inhibitors [28]. These findings suggest that circulating tumor‐derived exosomal signatures and ferroptosis biomarkers may provide novel tools for identifying patients at high risk of immune checkpoint inhibitor–associated cardiotoxicity and guiding preventive cardioprotective strategies.

Several limitations should be acknowledged. First, our study was conducted in vitro using primary cardiomyocytes and tumor‐conditioned media, which may not fully recapitulate the complexity of the in vivo tumor–immune–cardiac microenvironment. Additionally, while we focused on lung cancer–derived factors, other malignancies may exhibit distinct secretory profiles with varying cardiotoxic potential. Second, although we employed a comprehensive multi‐parametric panel to identify ferroptosis—including MDA, GPX4 suppression, GSH/GSSG depletion, labile iron accumulation, mitochondrial dysfunction, BODIPY C11 staining, and specific pharmacological rescue by ferrostatin‐1 and liproxstatin‐1—we acknowledge that ultrastructural validation by transmission electron microscopy (TEM) and direct immunofluorescence detection of lipid peroxidation adducts (e.g., 4‐HNE) were not performed due to technical constraints. Nevertheless, this biochemical and pharmacological approach aligns with current consensus guidelines for ferroptosis identification [29] and is widely accepted in high‐impact literature. Third, while our data demonstrate concurrent mitochondrial impairment and activation of NF‐κB/STING inflammatory signaling, the present experimental design does not establish temporal sequencing or direct causality between these events. Future studies employing in vivo tumor‐bearing models, patient‐derived samples, time‐resolved sampling, and targeted interventions (e.g., mitochondrial ROS scavengers, mtDNA‐release inhibitors, TEM, and 4‐HNE staining) will be essential to definitively map the mechanistic hierarchy, validate the clinical relevance of tumor‐derived ferroptotic priming, and develop predictive biomarkers for immune checkpoint inhibitor–associated cardiotoxicity.

**In conclusion**, our findings reveal a previously unrecognized mechanism of immune checkpoint inhibitor–associated cardiotoxicity driven by the synergistic interaction between tumor‐derived secretory factors and PD‐1 blockade. By identifying ferroptosis as a central node linking tumor–host communication, metabolic stress, mitochondrial dysfunction, and inflammatory signaling, this study provides a unifying framework for understanding cardiac injury during cancer immunotherapy. Targeting ferroptosis or its upstream regulators may offer novel strategies to preserve cardiac function without compromising antitumor efficacy.

## Author Contributions

**Xiaohua Ma:** conceptualization, software, investigation, writing – original draft. **Chenhui Liu:** data curation, visualization, project administration, writing – review and editing. **Xiufeng Li**, **Fuchao Qu**, **Hao Cheng**, **Yifei Liu:** data curation, review and editing.

## Funding

The authors have nothing to report.

## Ethics Statement

The study protocol was reviewed and approved by the Institutional Ethics Committee of the Fourth People's Hospital of Jinan (Approval No. LL20260017). All experimental procedures involving animal‐derived cells were conducted in accordance with institutional guidelines and national regulations for the care and use of laboratory animals.

## Consent

Informed consent was not applicable as this study utilized commercially available and ethically isolated cell lines.

## Conflicts of Interest

The authors declare no conflicts of interest.

## Supporting information


**Figure S1:** Assessment of apoptotic cell death in cardiomyocytes under combined tumor‐derived factors and PD‐1 blockade. Primary neonatal murine ventricular cardiomyocytes were treated with Control medium, tumor‐conditioned media (TCM), anti‐PD‐1 antibody (10 µg/mL), or the combination (TCM + anti‐PD‐1) for 24 h. (a) Caspase activity assays for executioner caspases (caspase‐3/7), extrinsic pathway caspase (caspase‐8), and intrinsic pathway caspase (caspase‐9). Data are presented as fold increase relative to control (mean ± SEM, n = 3 independent experiments). (b) Flow cytometric analysis of apoptosis using Annexin V‐FITC/propidium iodide (PI) dual staining. Percentages indicate the proportion of cells in each quadrant.

**Figure S2:** Tumor‐derived exosomal miRNAs directly target GPX4 and Nrf2 in cardiomyocytes. (a) Sequence alignment showing the predicted binding site of mmu‐miR‐214‐3p within the 3'UTR of mouse Gpx4. (b) Results of the dual‐luciferase reporter assay. (c) Sequence alignment showing the predicted binding site of mmu‐miR‐23a‐3p within the 3'UTR of mouse Nrf2. (d) Results of the dual‐luciferase reporter assay for Nrf2. Data are presented as mean ± SD. ***p < 0.001.

## Data Availability

All data generated or analyzed during this study are included in this published article and its supplementary information files. Raw datasets, including qRT‐PCR cycles, Western blot images, and statistical outputs, are available from the corresponding author upon reasonable request.
